# CD73 complexes with emmprin to regulate MMP-2 production from co-cultured sarcoma cells and fibroblasts

**DOI:** 10.1186/s12885-019-6127-x

**Published:** 2019-09-12

**Authors:** M. Aoki, K. Koga, M. Miyazaki, M. Hamasaki, N. Koshikawa, M. Oyama, H. Kozuka-Hata, M. Seiki, B. P. Toole, K. Nabeshima

**Affiliations:** 1 Department of Pathology, Fukuoka University School of Medicine, 7-45-1 Nanakuma, Jonan-ku, Fukuoka, 814-0180 Japan; 2 Division of Cancer Cell Research, Kanagawa Cancer Center Research Institute, Yokohama, Japan; 3 Division of Cancer Cell Research, Institute of Medical Science, University of Tokyo, Tokyo, Japan; 4 Medical Proteomics Laboratory, Institute of Medical Science, University of Tokyo, Tokyo, Japan; 5 Regenerative Medicine & Cell Biology, Medical University of South Carolina, Charleston, USA

**Keywords:** Emmprin, CD73, MMP-2, Sarcoma, Fibroblasts

## Abstract

**Background:**

Interaction between cancer cells and fibroblasts mediated by extracellular matrix metalloproteinase inducer (emmprin, CD147) is important in the invasion and proliferation of cancer cells. However, the exact mechanism of emmprin mediated stimulation of matrix metalloprotease-2 (MMP-2) production from fibroblasts has not been elucidated. Our previous studies using an inhibitory peptide against emmprin suggested the presence of a molecule on the cell membrane which forms a complex with emmprin. Here we show that CD73 expressed on fibroblasts interacts with emmprin and is a required factor for MMP-2 production in co-cultures of sarcoma cells with fibroblasts.

**Methods:**

CD73 along with CD99 was identified by mass spectrometry analysis as an emmprin interacting molecule from a co-culture of cancer cells (epithelioid sarcoma cell line FU-EPS-1) and fibroblasts (immortalized fibroblasts cell line ST353i). MMP-2 production was measured by immunoblot and ELISA. The formation of complexes of CD73 with emmprin was confirmed by immunoprecipitation, and their co-localization in tumor cells and fibroblasts was shown by fluorescent immunostaining and proximity ligation assays.

**Results:**

Stimulated MMP-2 production in co-culture of cancer cells and fibroblasts was completely suppressed by siRNA knockdown of CD73, but not by CD99 knockdown. MMP-2 production was not suppressed by CD73-specific enzyme inhibitor (APCP). However, MMP-2 production was decreased by CD73 neutralizing antibodies, suggesting that CD73-mediated suppression of MMP-2 production is non-enzymatic. In human epithelioid sarcoma tissues, emmprin was immunohistochemically detected to be mainly expressed in tumor cells, and CD73 was expressed in fibroblasts and tumor cells: emmprin and CD73 were co-localized predominantly on tumor cells.

**Conclusion:**

This study provides a novel insight into the role of CD73 in emmprin-mediated regulation of MMP-2 production.

## Background

Interaction between cancer cells and stromal cells is essential for cancer invasion and metastasis, however, the mechanism of interaction remains unclear. Invasion and metastasis of cancer cells require remodeling of the matrix surrounding the cancer cells. Fibroblasts present in the stroma are mainly responsible for producing the matrix metalloproteinases (MMPs), which play a central role in remodeling of the matrix [1]. Extracellular matrix metalloproteinase inducer (emmprin), a protein expressed in most tumors, plays an important role in regulating production of MMPs from fibroblasts [1–6]. Emmprin is a multifunctional protein that is expressed in reproductive cells, brain, eyes, and muscle, and play roles in immunity and reproduction in addition to its role in cancer [6–9]. Emmprin is coded by a gene present on p13.3 of chromosome 19, and it has 10 exons. Emmprin is a member of the immunoglobulin (Ig) superfamily, with two extracellular immunoglobulin-like domains and three conserved asparagine (N)-glycosylation sites and a molecular weight of 31–65 kDa, depending on the extent of the glycosylation [1, 6, 7, 10].

The first extracellular Ig domain of emmprin is required for inducing MMPs production from fibroblasts; N-glycosylation is also essential for activity [1, 3, 11, 12]. Moreover, we have previously reported that MMP-2 production from fibroblasts is induced by emmprin’s first Ig domain (ECI) peptide, when substituted with chitobiose (the disaccharide with which N-glycosylation starts) [13]. Gelatinase A (MMP-2) is the most abundant MMP in the tumor stroma and contributes to tumor invasion [14, 15]. However, to date, emmprin receptors on fibroblasts have not been confirmed, and the detailed mechanism of regulation of MMP-2 production is unknown. Previously, we reported that synthetic peptides carrying a partial ECI construct of emmprin sequence without chitobiose could inhibit emmprin activity. Four peptides were synthesized, each consisting of 20–23 amino acids and corresponding to about one fourth of the full length ECI sequence of the emmprin molecule. Only the second peptide (emp#2), which contains a putative N-glycosylation site sequence, inhibited emmprin-stimulated production of MMP-2 in co-cultures of fibroblasts [16]. As a hypothetical mechanism of emp#2 peptide inhibition of MMP-2 production from fibroblasts, it was proposed that binding of other proteins with emmprin is inhibited in the presence of emp#2 peptide. In other words, our findings suggested the formation of a complex with homophilic or heterophilic cis- or trans- interactions between emmprin molecules within the plasma membrane or between emmprin and an unidentified cell surface molecule(s).

In this study, we identified new molecules which formed a complex with emmprin in co-cultures of tumor cells and fibroblasts and analyzed whether these molecules were involved in the regulation of MMP-2 production.

## Methods

### Cell culture

Epithelioid sarcoma cell line FU-EPS-1 and immortalized human dermal fibroblast ST353i was established in our laboratory [17, 18]. These cell lines were maintained in D-MEM/Ham’s F-12 (Wako, Tokyo, Japan) growth medium, supplemented with 10% fetal calf serum (FCS), streptomycin (50 μg/ml), and penicillin (50 U/ml).

### Co-culture experiments

Co-culture experiments were performed as previously described [9, 13, 19] using fibroblasts (ST353i) and tumor cells (FU-EPS-1). Inhibition experiments in co-cultures using anti-CD73 blocking antibody (7G2, Abcam, Cambridge, UK, 1100) were performed as described previously [16, 20]. For the co-culture experiments employing CD73 specific inhibitor adenosine 5′-(α,β-methylene) diphosphate (APCP), co-cultured cells were incubated at 37 °C for 24 h to 7 d with 20, 40 and 80 μM of APCP.

### BS3 crosslinking

FU-EPS-1 and ST353i were co-cultured at a ratio of 1:1. The conditioned medium was replaced with 0.2% lactalbumin enzymatic hydrolysate containing serum-free medium after three washes with serum-free medium on day 2 and day 5. The cells were incubated with 3 mM BS3 (bis (sulfosuccinimidyl)suberate) solution for 30 min at room temperature. After removing the BS3 solution, free primary amine (10 mM tris pH 7.4, 2 x protein inhibitor) was added for 5 min to stop the cross-linking reaction.

### Immunoblotting

SDS-PAGE and immunoblotting were performed using 4–15% gradient gel (Bio-Rad, Hercules, CA) and antibodies against emmprin (mouse monoclonal, R&D System, Flanders, NJ), anti-CD73 (rabbit monoclonal, Cell Signaling, Danvers, MA), MMP-2 (monoclonal antibody, Daiichi Fine Chemical, Toyama, Japan) and MT1-MMP (Millipore, Bedford, MA). After electrophoresis, the proteins were transferred to an immobilon membrane (Millipore). Non-specific sites were blocked using 5% dry fat milk in Tris Buffered Saline (TBS) at 37 °C for 1 h and the membrane was incubated overnight at 4 °C with the primary antibody. After washing with TBS-T (TBS containing 0.05% tween 20), the membrane was incubated for 1 h with peroxidase-conjugated secondary antibody. Color was developed with chemiluminescence reagents according to the manufacturer’s instructions (PerkinElmer,Waltham,MA).

### Immunoprecipitation

Cell lysates (1 ml) were incubated with 2 μg anti-emmprin antibody (goat polyclonal antibody, R&D System), or anti-CD73 antibody (rabbit monoclonal, Cell Signaling) for 3 h. Protein G Sepharose (GE Healthcare, Chicago, Illinois) was used to immunoprecipitate proteins linked to the primary antibody overnight at 4 °C. After five washes with lysis buffer, bound proteins were eluted with 2 x sample buffer and subjected to immunoblotting.

### Mass spectrometric analysis

Following complexation of emmprin with membrane proteins using cross-linker (BS3) in FU-EPS-1 cells, the lysates were subjected to immunoprecipitation and immunoblotting. Three bands detected in co-culture of tumor cells and fibroblasts (75–100 kDa, 100–140 kDa, and 220 kDa) and a single band (220 kDa) detected in tumor cells alone were analyzed (Additional file 1: Figure. S1A and B). Mass spectrometry (MS) analysis was performed using the excised sample (identical molecular weight bands which reacted with anti-emmprin antibody in BS3 treated and BS3 non-treated samples). Shotgun proteomic analyses were performed using a linear ion trap-orbitrap mass spectrometer (LTQ-Orbitrap Velos, Thermo Fisher Scientific, Waltham MA) coupled with a nanoflow LC system (Dina-2A, KYA Technologies, Tokyo, Japan) as previously described [21]. Proteins were identified by querying MS and MS/MS data against the RefSeq (National Center for Biotechnology Information) human protein database using Mascot (Matrix Science, London, UK).

### Transient knockdown using RNA interference

Small interference RNA (siRNA) sequences were used for transient knockdown of CD99, CD73 and emmprin mRNA. siRNAs targeting the CD99 (Invitrogen, Carlsbad, CA), CD73 (Invitrogen) and emmprin (Invitrogen, Carlsbad, CA) were transfected in FU-EPS cells using Lipofectamine transfection reagent in Opti-MEM media (Thermo Fisher Scientific) in the absence of serum and antibiotics according to the manufacturer’s instructions. Knockdown of CD99, CD73 and emmprin expression in cells was analyzed by immunoblotting. Each siRNA was transfected into fibroblasts and tumor cells separately, and transfected cells were used for co-culture experiments.

### Enzyme-linked immunosorbent assay (ELISA)

After 48 h of incubation, conditioned media were collected from ST353i alone or FU-EPS-1 and ST353i co-cultures and the expression of MMP-2 was measured using the Total MMP-2 Quantikine ELISA Kit (sensitivity: 0.082 ng/ml) according to the manufacturer’s specifications (R&D Systems, Minneapolis, MN). All assays were performed in triplicate and statistical analyses were performed using Student’s t-test.

### Zymography

Gelatinolytic activities in conditioned media were demonstrated using gelatin as a substrate, as described previously [19, 20]. We measured the enzymatic activity of pro-MMP-2 using the commercially available Gelatin-Zymography kit (Cat.No.AK47-COS, Cosmo Bio, Tokyo) by following the protocols supplied by the manufacturer. The enzyme activity was detected as a clear band on the resulting blue background of undigested gelatin.

### In situ proximity ligation assay (PLA)

In situ PLA was used to assess protein-protein interactions. Cells grown on 8-well culture slides (Nunc Lab-Tek chamber Slide System, ThermoFisher scientific), were immediately fixed and subjected to in situ PLA using the Duolink detection kit (Sigma-Aldrich, St. Louis, MO) according to the manufacturer’s instructions and described previously [22]. After blocking, slides were incubated with mouse anti-Emmprin (1:100; R&D) or rabbit anti-CD73 (1:100; GeneTex, Irvine, CA) primary antibodies. For isotype controls, the primary antibody was substituted with either mouse (emmprin) or rabbit (CD73) IgG. To analyze the results, we used a Duolink ImageTool to obtain objective quantification of PLA signals.

### Immunohistochemistry

Four-micrometer thick paraffin-embedded sections were deparaffinized and heated in a microwave oven (700 watts) for 10 min to expose antigens in a 10 mM Na-citrate buffer, followed by blocking in 3% hydrogen peroxide solution. The sections were incubated with anti-emmprin (mouse monoclonal, R&D, 1:200) and CD73 (rabbit polyclonal, Abcam, 1:100) antibodies at 4 °C overnight. The exposed antigen was detected using EnVision reagent conjugated horseradish peroxidase (Agilent, Santa Clara, CA). The reaction was identified with DAB and counterstained with Mayer’s hematoxylin. The staining results were evaluated semi-quantitatively by two independent observers. Immunostaining was considered negative if the percentage of stained tumor cells or stromal cells was < 10%. In specimens considered positive, staining of the cells was quantified on a scale of 1–4 based on the percentage of positive cells. The scale was structured as follows: 1+, 10–25% of cells positive; 2+, 25–50% of cells positive; 3+, 50–75% of the cells positive; 4+, > 75% of the cells positive. CD73-close and CD73-distant staining score were means of three high power fields of view. CD73-close included stromal cells positioned close to the tumor cells, and CD73-distant were those most distant from the tumor cells in the same slides.

### Immunofluorescence

Tumor cells and fibroblasts grown on 8-well culture slides for 48 h were fixed in 4% paraformaldehyde at 4 °C for 30 min, and permeabilized with 0.1% Triton X-100 in phosphate-buffered saline. After blocking with Image-iT FX Signal Enhancer (Invitrogen) for 1 h at room temperature, the slides were incubated with anti-emmprin (mouse monoclonal, R&D; 1:100) and anti-CD73 (rabbit polyclonal, abcam; 1:100) antibodies at 4 °C overnight. Secondary antibodies and mounting medium were anti-mouse IgG Alexa594 (Invitrogen), anti-rabbit IgG Alexa488 (Invitrogen), and Mounting Medium with DAPI (Abcam). Images of cells were captured using an Biozero BZ-8000 (Keyence, Osaka, Japan).

### Statistical analysis

Quantitative data are presented as mean ± standard deviation (SD) and were analyzed using the Student’s t test. A *p* value < 0.01 was considered indicative of statistical significance.

### Tissue samples

The study material comprised 10 epithelioid sarcoma samples from two males and eight female patients (age range: 22–81 y; mean: 61 y) obtained from the soft tissue tumor file of the Department of Pathology, Fukuoka University Hospital, between 1995 and 2015. Use of anonymous and redundant tissue is part of the standard treatment agreement with patients in our hospital when no objection has been expressed.

## Results

### Identification of molecules which form a complex with emmprin by MS analysis

**Fig. 1:**
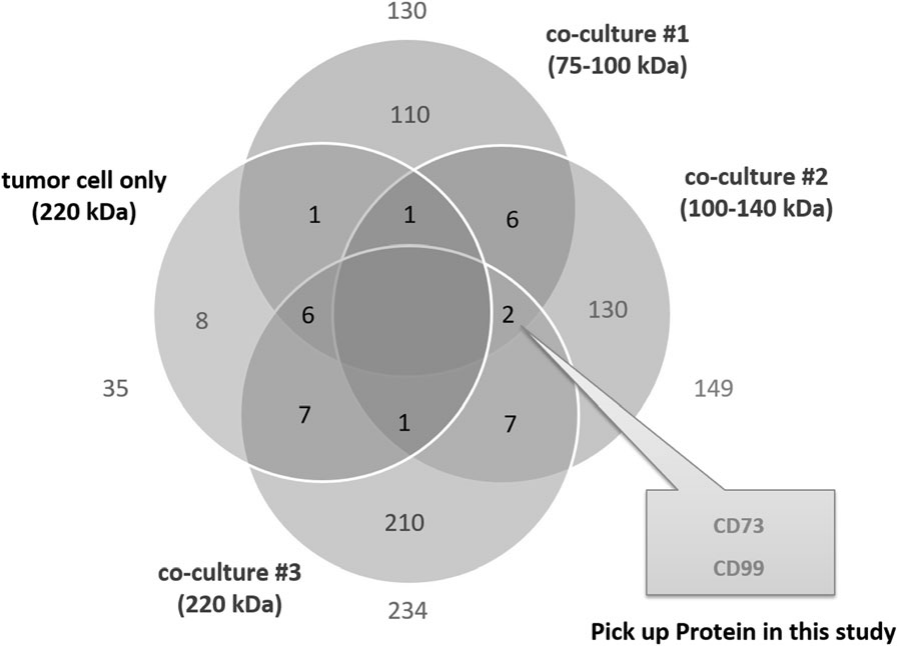
MS analysis of emmprin complexes identified CD73 and CD99. Proteins forming complexes with emmprin were identified from cancer cells alone or from co-cultures of cancer cells and fibroblasts, and were analyzed by immunoprecipitation, cross-linking, and mass spectrometric (MS) protein identification. A total of 548 protein molecules were identified using MS. Overlap between proteins identified in different conditions (tumor cell only or three molecular weight regions under co-culture conditions #1–3) is shown. CD73 and CD99 identified in the overlap of all three co-culture conditions were selected for investigation of their effect on regulation of MMP-2 production

Proteins extracted from co-culture of tumor cells and fibroblasts, that had been cross-linked with BS3, were immunoprecipitated using anti-emmprin antibody and subjected to immunoblotting. Proteins from molecular weight regions of 75–100, 100–140, and 200 kDa were extracted from the gel (Additional file 1: Figure. S1A) and subjected to MS analysis. A total of 130, 149, and 234 proteins were identified by MS analysis in these molecular weight regions. Overlap in the proteins obtained by a total of four MS analyses, including the proteins from tumor cells alone (molecular weight region of 220 kDa, Additional file 1: Figure. S1B) analyzed in the same way, is shown in Fig. 1. Emmprin was detected in all four analyses. CD73 and CD99, detected in all three analyses of co-cultured tumor cells and fibroblasts, were the target proteins selected for this study.

### Both CD73 and CD99 form a complex with emmprin

**Fig. 2:**
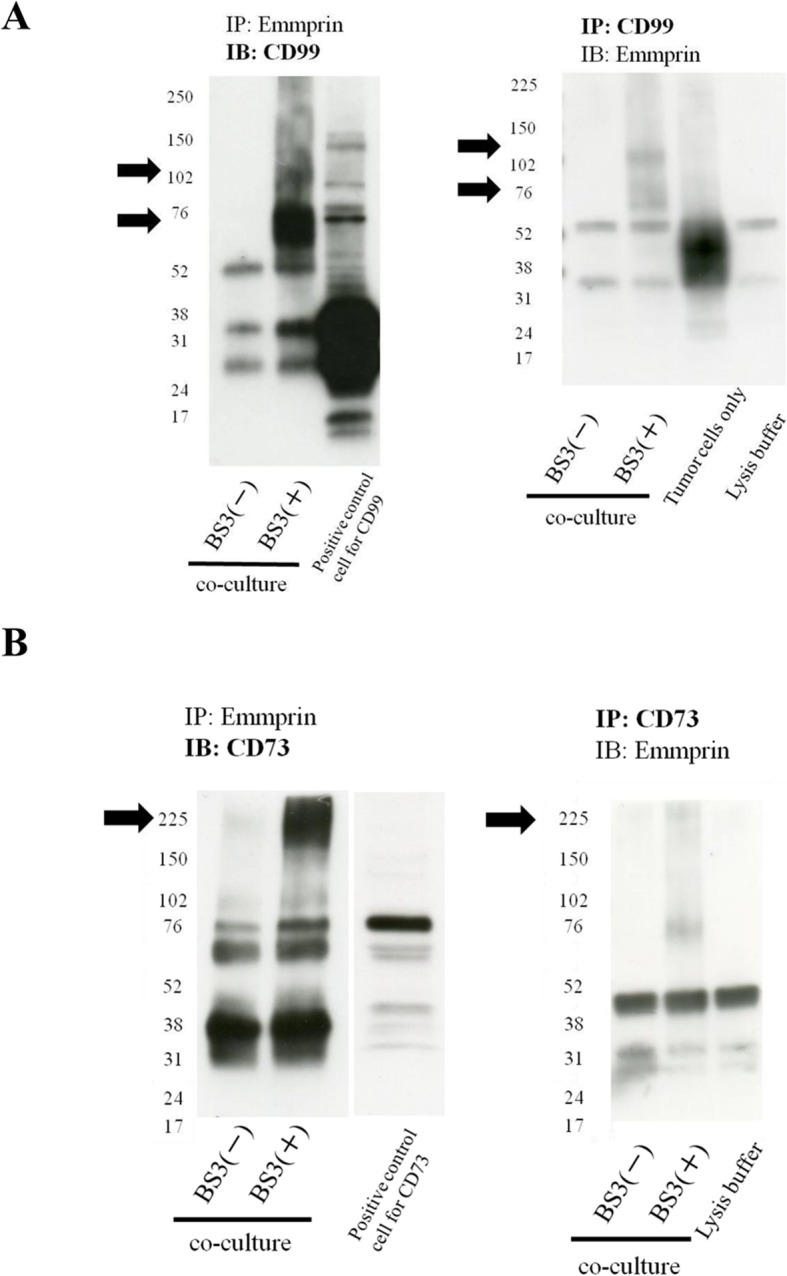
CD99 and CD73 form complexes with emmprin. **a** Complex formation between emmprin and CD99 was identified by immunoprecipitation and immunoblotting, performed in co-cultures of tumor cells and fibroblasts. Arrow indicates emmprin-CD99 complex. **b** Complex formation between emmprin and CD73 was identified by immunoprecipitation and immunoblotting, performed in co-cultures of tumor cells and fibroblasts. Arrow indicates emmprin-CD73 complex.

Complex formation with emmprin was confirmed by using immunoprecipitation and immunoblotting. When immunoprecipitation was performed with emmprin or CD99 antibodies, and immunoblotting was carried out with either one of these antibodies, unique bands in the lane treated with BS3 were noted in the vicinity of 102 kDa and 76 kDa (Fig. 2a), suggesting the formation of an emmprin and CD99 complex. When immunoprecipitation was carried out in the same way with emmprin or CD73 antibodies, and immunoblotting was carried out with either one of these antibodies, a unique band in the lane treated with BS3 was noted in the vicinity of 225 kDa (Fig. 2b), indicating the formation of a complex of emmprin and CD73.

### CD73 and CD99 are expressed in both tumor cells and fibroblasts

**Fig. 3:**
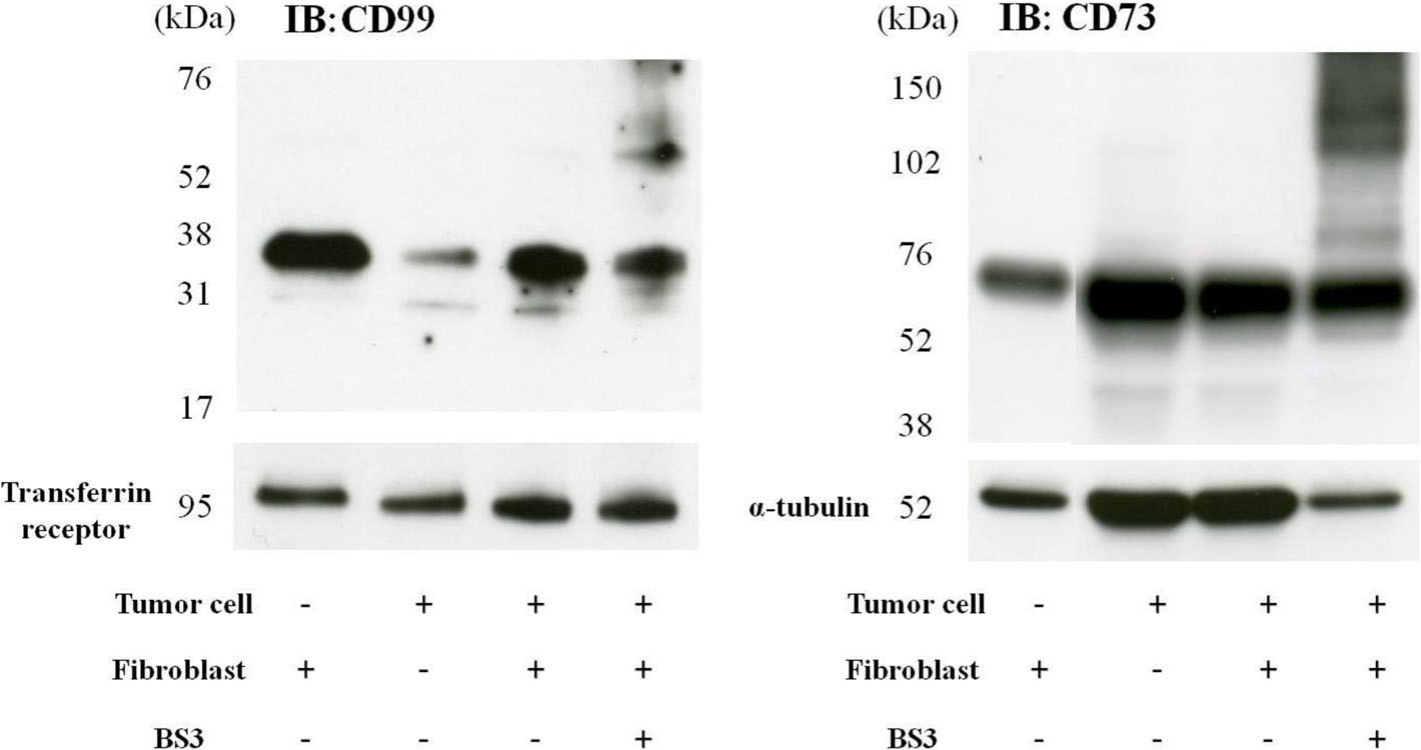
CD99 and CD73 expression in tumor cells, fibroblasts and their co-culture. Both CD99 and CD73 were expressed in tumor cells and fibroblasts. CD99 was expressed at a higher level in fibroblasts than tumor cells. CD73 showed equivalent expression in fibroblasts (ST353i) and tumor cells (FU-EPS-1) CD99 (32 and 25 kDa), protein was extracted from membrane preparations; CD73 (70 kDa) protein was extracted from total cell lysate.

Using immunoblotting, CD99 and CD73 were expressed by both tumor cells and fibroblasts, when cultured separately (Fig. 3). CD99 showed high expression in fibroblasts. No apparent increase was observed in expression of CD99 or CD73 upon co-culture of the tumor cells and fibroblasts.

### CD73 regulates the production of MMP-2 from co-cultures of sarcoma cells with fibroblasts

**Fig. 4:**
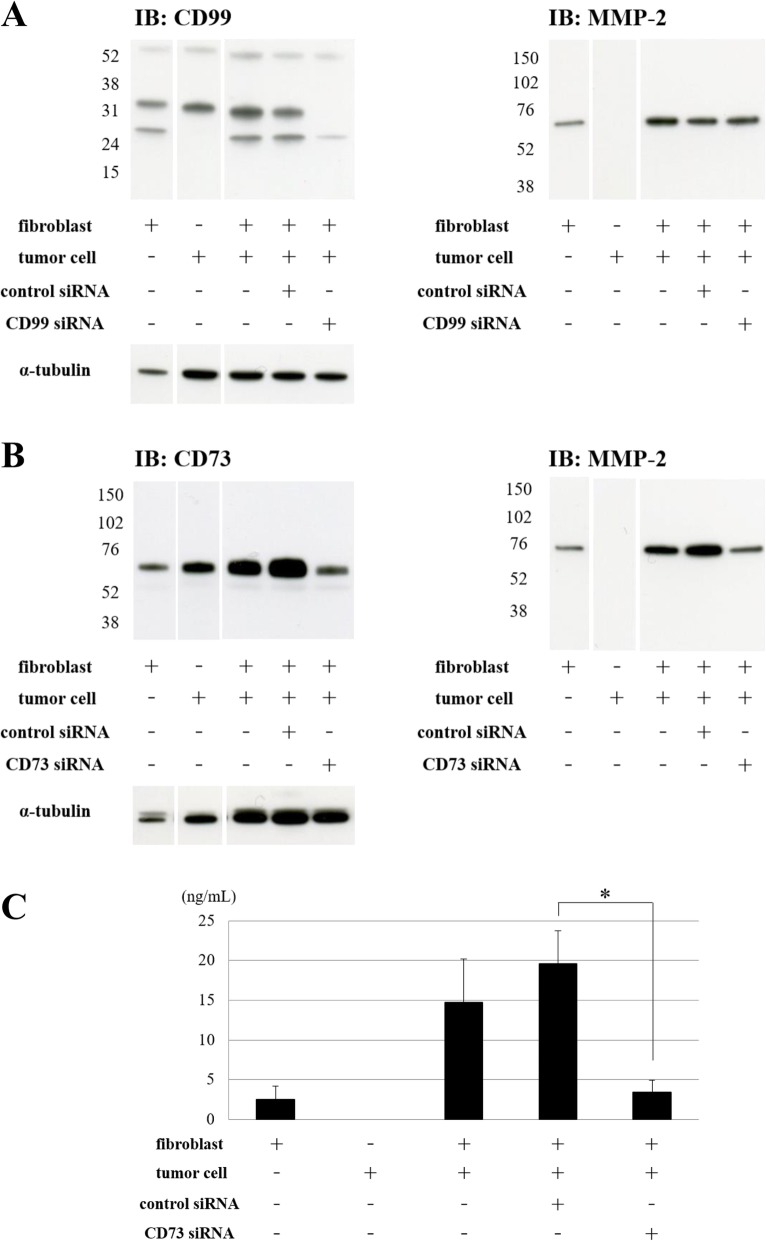
Knockdown of CD73 in co-culture or fibroblasts reduced of MMP-2 production from fibroblasts. **a** CD99 siRNA (0.02 pmol/μl) treatment causes knockdown of CD99 expression (32 and 25 kDa) in co-culture of tumor cells (FU-EPS-1) with fibroblasts (ST353i). Protein was extracted from membrane preparation. No reduction of MMP-2 production in conditioned medium in co-cultures of tumor cells with fibroblasts was observed upon siRNA mediated CD99 knockdown in co-cultured cells. **b** CD73 siRNA (0.01 pmol/μl) treatment causes knockdown of CD73 expression in co-culture of tumor cells (FU-EPS-1) with fibroblasts (ST353i). Protein was extracted from whole cell lysate. Reduction of MMP-2 production in conditioned medium was observed upon siRNA mediated CD73 knockdown in co-cultures of tumor cells with fibroblasts. **c** Enzyme-linked immunosorbent assay (ELISA) of MMP-2 in conditioned medium of fibroblasts, tumor cells, and co-cultured cells. These co-cultured cells were transfected with CD73-specific or control siRNA. MMP-2 production from co-cultured cells decreased significantly in CD73 transfected cells. *p < 0.01

Experiments using siRNA were performed to investigate whether CD99 and CD73 were associated with the regulation of MMP-2 production from co-cultures of sarcoma cells with fibroblasts. Expression of CD99 and CD73 was suppressed using CD99 or CD73 siRNA in co-cultures of sarcoma cells with fibroblasts (Fig. 4a-c). MMP-2 was not detected in the cell supernatant of tumor cells alone, and the expression of MMP-2 in the supernatant during co-culture was increased, compared with that in the culture of fibroblasts alone. No suppression in MMP-2 expression was observed when CD99 was suppressed by using siRNA. However, MMP-2 production in the supernatant was suppressed in co-culture, by suppressing the expression of CD73 via siRNA (Fig. 4b). MMP-2 production in co-cultures of tumor cells with fibroblasts were also suppressed by emmprin siRNA induction (Additional file 2: Figure. S2). MMP-2 production was also determined by using ELISA (Fig. 4c). As in the results of immunoblotting, MMP-2 production from the fibroblasts detected by ELISA was increased during co-culture, compared with that in the culture of fibroblasts alone, and production of MMP-2 from co-culture was significantly decreased by using CD73 siRNA. These results showed that CD73 regulates MMP-2 production from fibroblasts. These 68-kDa MMP-2 band in the Fig. 4 corresponds to the molecular mass of the pro-form of MMP-2 [23], and we also confirmed the pro-MMP-2 gelatinolytic band using zymography (Additional file 5: Figure. S5A). On the other hand, the tumor cells expressed membrane-type1 MMP (MT1-MMP), the physiological activator of proMMP-2 (Additional file 5: Figure. S5B).

### CD73 is co-expressed and co-localized with emmprin

**Fig. 5:**
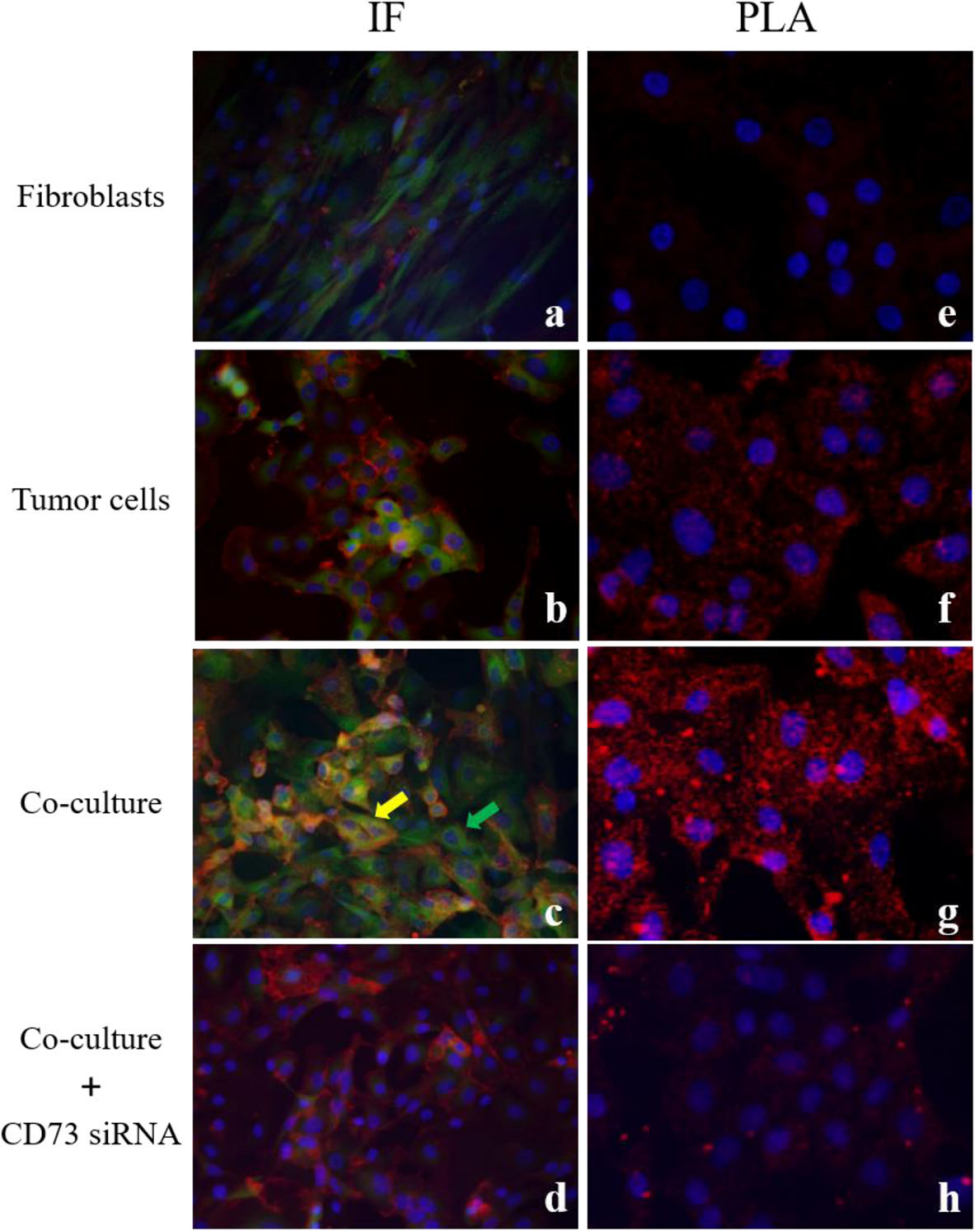
Colocalization of emmprin/CD73 detected by immunofluorescent staining and in situ proximity ligation assay. Cytoplasmic CD73 (green) expression was observed in fibroblasts, tumor cells and co-culture cells. Membranous emmprin expression (red) was observed in tumor cells and co-cultured cells. Nuclei were stained with DAPI (blue). CD73 and emmprin were colocalized in tumor cells (**b**) and co-cultured cells (**c**). The green arrow points to fibroblasts expresssing green florescence (CD73); The yellow arrow points to tumor cells expressed yellow florescence (emmprin and CD73) (**c**). CD73 siRNA treatment causes downregulation of CD73 cytoplasmic expression, although, membranous emmprin expression was retained in co-cultured cells (**d**). The fluorescent red spots observed using in situ proximity ligation assay (PLA), indicating protein-protein colocalization in cells, confirmed the interaction between CD73 and emmprin. The detected dimers (emmprin/CD73) are represented as red dots in co-cultured cells (**g**). In cells transfected with CD73 siRNA prior to in situ PLA for emmmprin-CD73 interaction, CD73 siRNA treatment caused downregulation of red dots (**h**). Immunofluorescent staining, IF (**a**-**d**); In situ proximity ligation assay, PLA (**e**-**h**); fibroblast only (**a**, **e**); tumor cell only (**b**, **f**); co-culture (**c**-**d**, **g**-**h**)

With respect to the localization of CD73 and emmprin, the possible co-expression of these molecules on the cell was analyzed using fluorescent immunostaining. Fibroblasts showed predominant CD73 expression with faint emmprin expression, as detected by fluorescent immunostaining (Fig. 5a). In the tumor cells and in co-culture, emmprin was expressed on the membrane, and expression of CD73 was seen in both the cytoplasm and membrane; expression of both proteins was observed on the same cells (Fig. 5b, c). Green arrows indicate fibroblasts only expressing green florescence (CD73), while yellow arrows point to tumor cells expressing yellow, which are a mixed florescence signal of red florescence (emmprin) and green florescence (CD73). Upon suppressing the expression of CD73 by siRNA, only the expression of emmprin remained on the cell membrane (Fig. 5d).

A PLA signal is detected as a red dot when the recognition sites of two antibodies are in close proximity, making it possible to verify whether two proteins are co-localized. In our case, PLA experiments were performed using antibodies for CD73 and emmprin. With fibroblasts alone, there was no observable signal (Fig. 5e). A PLA signal was detected with tumor cells alone (Fig. 5f) and under co-culture conditions (Fig. 5g). Furthermore, a much stronger signal was obtained under co-culture conditions than with tumor cells alone. When CD73 was suppressed by siRNA, PLA signals were inhibited (Fig. 5h). The PLA signals were quantified by using Image Tool analysis, which confirmed that the signal intensity was clearly decreased by CD73 siRNA vs. control siRNA (Additional file 3: Figure. S3).

These results show that in tumor cells or in co-cultured fibroblasts and tumor cells, CD73 and emmprin are co-expressed and co-localized.

### CD73 suppresses MMP-2 production via non-enzymatic activity

**Fig. 6:**
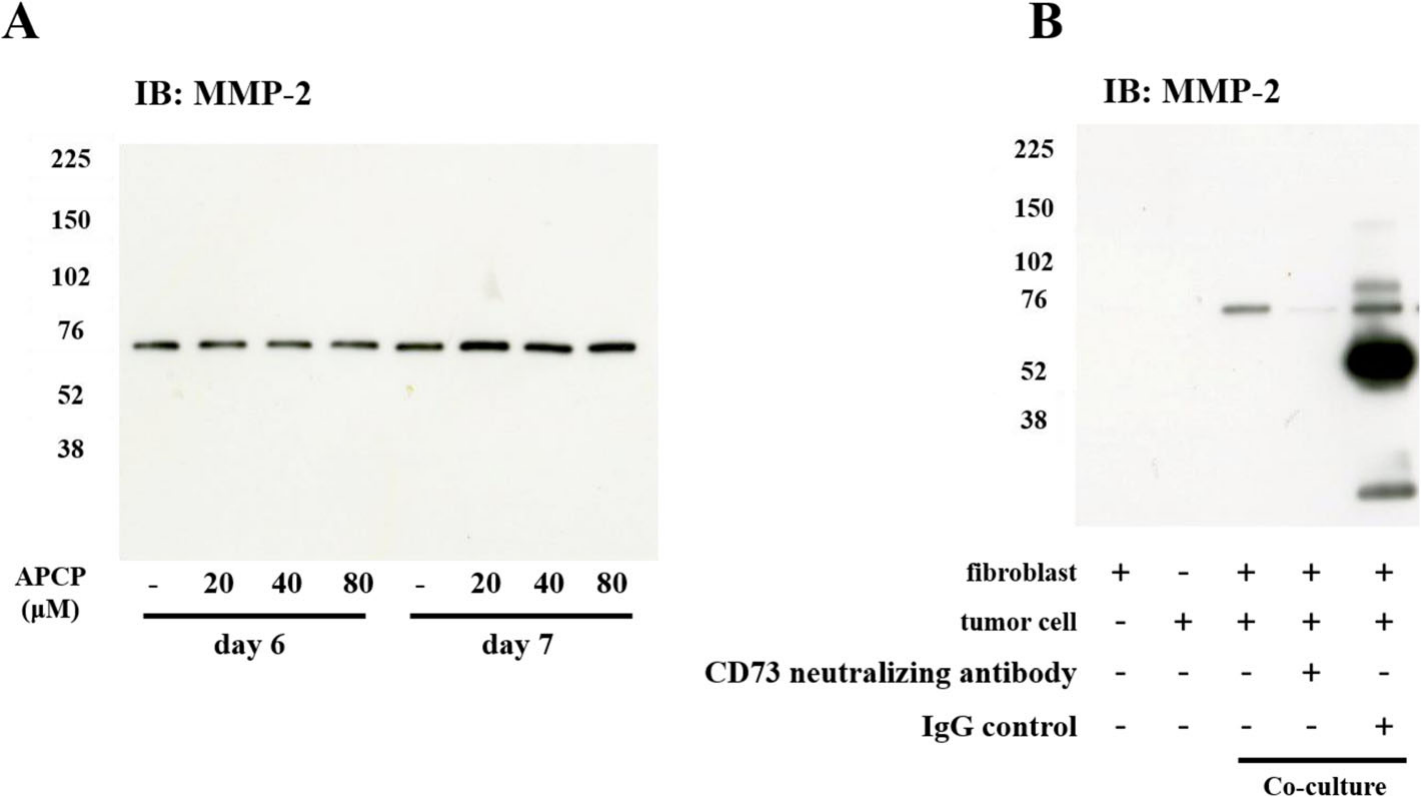
Effects of CD73 specific inhibitor APCP and CD73 neutralizing antibody on MMP-2 production in co-cultured cells. **a** MMP-2 production in conditioned medium from co-cultured cells was not reduced by addition of 20–80 μM CD73 specific inhibitor adenosine 5′-(α,β-methylene) diphosphate (APCP). **b** Use of CD73 neutralizing antibody demonstrates apparent inhibition of MMP-2 production in conditioned medium from co-cultured cells, while control IgG did not cause any inhibition

CD73 has enzyme activity to convert adenosine monophosphate (AMP) to adenosine. CD73 also possesses non-enzymatic activity [24, 25]. We investigated whether the enzymatic activity of CD73 is responsible for the suppression of production of MMP-2. α,ß-methyleneadenosine 5′-diphosphate (APCP) is a specific inhibitor of CD73, which specifically inhibits CD73 enzymatic activity to converting ADP to adenosine. Co-cultured fibroblasts and tumor cells were treated with APCP at a concentration of 20–80 mM. No differences were seen in the levels of MMP-2 in the cell supernatant in the presence or absence of APCP (Fig. 6a). By contrast, upon adding anti-CD73 neutralizing antibodies to co-cultured fibroblasts and tumor cells, MMP-2 production in the supernatant was clearly suppressed, compared with the control (Fig. 6b). These data show that MMP-2 production from fibroblasts is suppressed by anti-CD73 neutralizing antibodies (Fig. 6b) and CD73 siRNA (Fig. 4b, and c), and not by APCP (Fig. 6a), indicating that suppression of MMP-2 production by CD73 is via its non-enzymatic activity.

### Co-expression of emmprin and CD73 in vivo

**Fig. 7:**
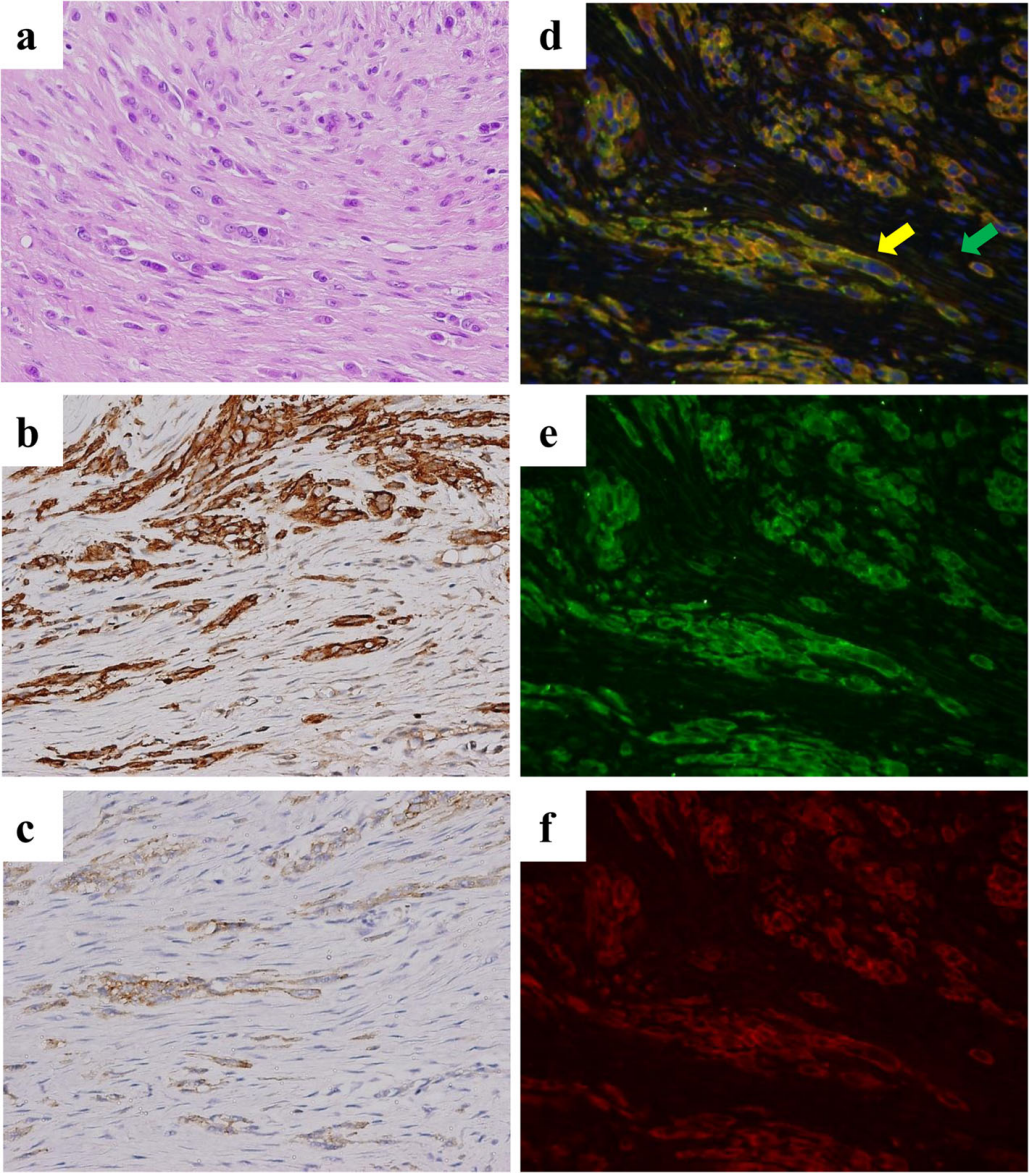
Expression of CD73 and emmprin in epithelioid sarcoma. The hematoxylin and eosin (H&E) section shows proliferation of severely atypical polygonal cells with enlarged hyperchromatic nuclei, forming irregular nests, accompanied by fibroblastic cells and fibrous stroma (**a**). Immunohistochemical (**b**-**c**) and fluorescent immunohistochemical (**d**-**f**) expression of CD73 and emmprin in epithelioid sarcoma specimen was examined. Both tumor cells and surrounding stromal cells were positive for CD73 (**b**). Membranous emmprin expression was observed only in tumor cells (**c**). Cytoplasmic CD73 (green) expression was observed in fibroblasts and in tumor cells (**e**). Membranous emmprin expression (red) was observed in tumor cells (**f**). Marger of figures (**e**) and (**f**). The green arrow, indicates fibroblasts expressing green florescence (CD73); The Yellow arrow, indicates tumor cells expressing yellow florescence (emmprin and CD73) (d). CD73 and emmprin were colocalized in tumor cells (**e**). Nuclei were stained with DAPI (blue). Expression of CD73 and emmprin was examined immunohistochemically in a total of ten tumors. All tumors show similar expression pattern (Additional file 6: Table S1)

In vivo expression of emmprin and CD73 was investigated by using immunohistochemistry and fluorescent immunostaining. Staining was performed using paraffin-embedded sections obtained from cases of epithelioid sarcoma patients. Epithelioid sarcoma is characterized by the proliferation of epithelioid tumor cells with enlarged round to oval nuclei, accompanied by proliferation of surrounding stromal cells that are mainly fibroblasts (Fig. 7a). Immunohistochemistry and immunofluorescent immunostaining data shows that the cytoplasm and membrane of tumor cells and the stromal cells surrounding the tumor cells were both positive for CD73 (Fig. 7b, and e) and that the membrane of the tumor cells were positive for emmprin; however emmprin was not expressed in the stromal cells (Fig. 7c and f). Double immunofluorescence immunostaining confirmed that emmprin and CD73 are co-expressed on the tumor cells. The green arrow indicated fibroblasts only expressing green florescence (CD73), while the yellow arrow indicates tumor cells expressing yellow, which is a mixed florescence signal of red florescence (emmprin) and green florescence (CD73) (Fig. 7d). Similar results were obtained in all immunostained tumors. Overall, emmprin and CD73 were both highly expressed in tumor cells. Furthermore, high expression of CD73 was seen in the stromal cells surrounding the tumor cells (Additional file 6: Table S1, Additional file 4: Figure. S4). These results show that, as observed in in vitro results, emmprin and CD73 are also co-expressed in tumor cells in vivo.

## Discussion

In this study, we have for the first time reported that CD73 forms a complex with emmprin and regulates the production of MMP-2 from fibroblasts, although there has been a previous report providing evidence that suppression of CD73 leads to down-regulation of MMP-2 [26]. Findings of CD73 complexation with emmprin were confirmed by immunoprecipitation, double immunofluorescence staining, and proximity ligation assay. CD73 siRNA and CD73 neutralizing antibody were used to confirm that CD73 regulates MMP-2 production in co-cultures of sarcoma cells with fibroblasts.

Emmprin acts as a receptor for cyclophilins, S100A9 and platelet glycoprotein VI [10, 27], and also associates tightly with monocarboxylate transporters [7, 10, 28, 29]. Emmprin forms complexes with GLUT1, CD44 and CD98 on the cell membrane [10, 30]. Caveolin-1 [31, 32], MT1-MMP/MMP-14 [33], AnxA2 [34, 35], and integrins [36] have also been identified as binding partners in interactions with emmprin, and are thereby suggested to be involved in the regulation of MMP activity. However, it is possible that there are other emmprin binding partners which have not hitherto been reported.

We performed MS analysis to identify the proteins that bound with emmprin when tumor cells were co-cultured with fibroblasts. CD73 and CD99, detected by overlap in MS analyses of three different co-culture experiments, were selected and each protein was further analyzed (Fig. 1). The fact that these proteins were detected as conserved proteins in the analysis of three different co-culture experiments indicated that CD73 and CD99 were the proteins that bound most reproducibly with emmprin under conditions of co-culture of tumor cells and fibroblasts. Indeed, CD73 and CD99 were expressed abundantly both in fibroblasts and in tumor cells (Fig. 3).

CD73 is also known as ecto-5′-nucleotidase (ecto5′-NT). It is mainly present on cell membranes and functions as an enzyme which converts AMP to adenosine; it also has non-enzymatic activity [24, 25]. CD73 has been shown to be expressed in tumor cells and in the stroma of colorectal cancer [37, 38], prostate cancer [39, 40], gastric cancer [41], breast cancer [42], ovarian cancer [43] and squamous cell carcinoma [44]. Whether high expression of CD73 in cancer predicts poor prognosis or favorable prognosis is controversial, with published reports supporting both outcomes [25, 37–40, 42, 44]. According to our literature review, the expression of CD73 in sarcoma and stromal cells of sarcoma has not been previously reported. In this study, APCP, an inhibitor of CD73’s enzymatic activity did not inhibit MMP-2 production from co-culture cells from one (data not shown) to 7 days in a concentration of 20–80 μM (Fig. 6a). These results signify that inhibition of CD73 enzymatic activity does not effect MMP-2 production from the co-culture cells. These results support the possibility of the importance of emmprin-CD73 interaction that is needed for MMP-2 production, in that CD73 inhibition by siRNA and neutralizing antibody induced the suppression of MMP-2 production from co-cultured cells (Fig. 6b). The possibility cannot be denied that CD73 neutralizing antibody may disturb the interaction of CD73 and emmprin.

**Fig. 8:**
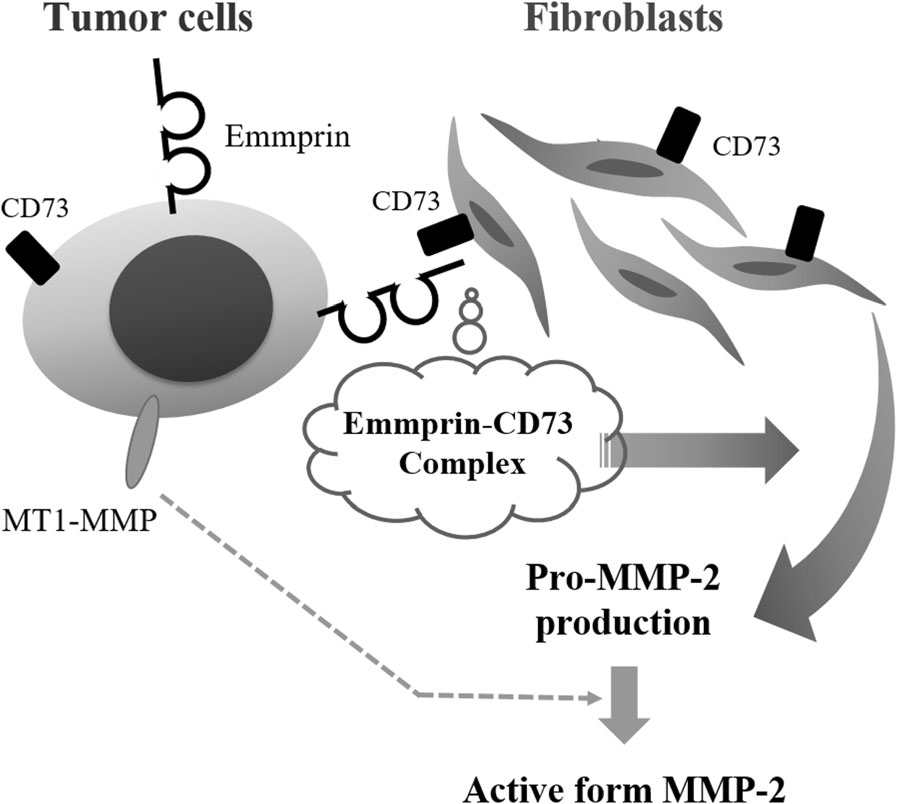
A pictorial representation of the interaction between emmprin on tumor cells and CD73 on fibroblasts. Emmprin mainly exists on tumor cells, and CD73 exists both on tumor cells and fibroblasts. Emmprin forms a complex with CD73, and regulates MMP-2 production in co-cultures of tumor cells and fibroblasts. Pro-MMP-2 produced by fibroblasts is probably activated by MT1-MMP expressed on tumor cells

Both CD73 and emmprin were expressed on tumor cells. However, PLA analysis revealed a much stronger signal under co-culture conditions compared with those on tumor cells cultured alone. Thus, trans-manner complex formation between CD73 on fibroblasts and emmprin on tumor cells possibly regulates MMP-2 production from fibroblasts. MMP-2 produced by fibroblasts is pro-form, which is probably activated by MT1-MMP expressed on tumor cells (Additional file 5: Figure. S5B), leading to promotion of the tumorigenesis of cancer cells (Fig. 8).

In this study, we analyzed the role of CD73 in cancer using an epithelioid sarcoma cell line and in vivo specimens taken from cases of epithelioid sarcoma, which is a highly aggressive non-epithelial neoplasm. Epithelioid sarcoma is a very rare tumor; however, the tumor cells exhibit an epithelioid morphology. It is therefore relatively easy to distinguish tumor cells from stromal cells in epithelioid sarcoma specimens histologically (Fig. 7). In these in vivo cases, emmprin was expressed in tumor cells and CD73 expression was observed in tumor cells as well as in fibroblasts (Fig. 7, Additional file 6: Table S1). CD73 was more highly expressed in the stromal fibroblasts close to the tumor cells, although the difference was not statistically significant (Additional file 6: Table S1, Additional file 4: Figure. S4). As we used biopsy samples, stromal CD73 expression of CD73-close and -distant were examined in the same samples: this was because, during the biopsies, no tissue suitable for use as a control were collected. It is necessary to examines CD73 expression in fibroblasts more distant from the tumor cells in the other tumor specimens. CD73 is expressed in both tumor cells and fibroblasts and emmprin is mainly expressed in tumor cells as shown in in vitro (Figs. 3 and 5) and in vivo (Fig. 7). Co-expression of emmprin and CD73 were observed mainly in tumor cells by fluorescent immunostaining (arrows in Figs. 5 and 7). These results suggest that CD73 and emmprin form a complex at the adhesion site of the tumor cells and adjacent fibroblasts. These findings also suggest the possibility of tumor-stromal interaction associated with CD73 in stromal cells.

CD99 is a transmembrane glycoprotein of 25 kDa and 32 kDa, coded by the gene MIC2 in the short arms of the X and Y chromosomes. CD99 is a marker of Ewing sarcoma and primitive neuroectodermal tumors. Its expression has been confirmed in malignant tumors of hematopoietic neoplasm, synovial sarcoma, solitary fibrous tumors, testicular, ovarian sex cord tumors, and breast cancer [45, 46]. Ours is the first study to demonstrate formation of a complex between CD99 and emmprin. While the CD99-emmprin complex was detected, inhibition of CD99 expression did not suppress MMP-2 production by fibroblasts. The precise function of the CD99 and emmprin complex is, as yet, unclear. Both CD99 and CD73 were expressed in tumor cells as well as in fibroblasts, although, more abundant expression was observed in fibroblasts than in tumor cells (Fig. 3). Previously, it has been reported that the expression of CD99 in lung cancer tumor stroma is associated with poor prognosis [45], which, together with our observation of CD99 with emmprin, suggests the possibility that CD99 also contributes to an interaction between tumor and stroma.

Expression of emmprin has been confirmed in many malignant tumors, and it has been reported to be associated with the progression of cancer [6]. In some sarcomas, a correlation between emmprin expression in sarcoma cells and prognosis has also been reported [18–20]. Therapeutic options are limited, especially with advanced sarcoma compared to epithelial neoplasms. Our study demonstrated a new insight into the mechanism of sarcoma invasion, that may possibly become a target of therapy.

This study had the following limitations. We demonstrated the complex formation and co-expression of CD73 and emmprin; further, we showed that CD73 was associated with the regulation of MMP-2 production in co-cultures of sarcoma cells with fibroblasts. However, we did not demonstrate directly whether the complex formation of CD73 and emmprin is essential for the regulation of MMP-2 production in co-cultures of sarcoma cells with fibroblasts. We performed this study with the sarcoma cell line FU-EPS-1. However, the finding that emmprin-CD73 interaction regulates MMP-2 production in co-cultures of tumor cells with fibroblasts has also been confirmed using squamous cell carcinoma cell lines obtained from head and neck tumor (data not shown). The function of CD99, which also forms a complex with emmprin, likewise remains unsolved. Clarification of these unclear points will be addressed in further detailed studies.

## Conclusions

In conclusion, using a combination of in vitro co-culture assays, mass spectrometry, and biological/molecular analysis, we demonstrated that CD73 forms a complex with emmprin to regulate the production of MMP-2 from fibroblasts. In vivo studies performed in tumor tissue sections confirmed colocalization of CD73 and emmprin. Inhibition of CD73 by either a neutralizing antibody or an siRNA, suppressed MMP-2 production from fibroblasts. Our study provides a novel insight into the role of CD73 in emmprin-mediated cancer invasion and metastasis.

## Supplementary information

**Additional file 1:Figure. S1.** Proteins isolated from membrane preps were subjected to silver staining in SDS-polyacrylamide gels (left) and immunoblotting (right). Mass spectrometry (MS) analysis was performed on the excised gel samples (identical molecular weight bands detected with anti-emmprin antibody in BS3 treated and BS3 non-treated samples). Three bands detected in the co-culture of tumor cells and fibroblasts shown in A (arrow #1, 75–100 kDa; arrow #2, 100–140 kDa; and arrow #3, 220 kDa) and a single band detected in tumor cells alone shown in B (arrow head, 220 kDa) were analyzed. (PDF 64 kb)

**Additional file 2:Figure. S2.** Emmprin siRNA (0.02 pmol/μl) treatment causes knockdown of emmprin expression (40-60 kDa) in co-culture of tumor cells (FU-EPS-1) with fibroblasts (ST353i). Protein were extracted from a membrane preparation. Reduction of MMP-2 production in the conditioned medium was observed upon siRNA mediated emmprin knockdown of co-cultured cells. (PDF 45 kb)

**Additional file 3: Figure. S3.** Quantification of PLA signals was performed by Image tool analysis (Duolink). (PDF 14 kb)

**Additional file 4: Figure. S4.** Representative CD73 expression in the stromal fibroblasts (arrow). CD73-close, close to the tumor cells scale 3+; CD73-distant, distant from the tumor cells scale 1 + . (PDF 138 kb)

**Additional file 5: Figure. S5.** A. MMP-2 gelatinolytic activity in fibroblasts and co-culture. Gelatin zymography was performed with culture media collected on day 7 of culture. Bands at 68 kDa correspond to the pro-form of MMP-2. Lane 1, MMP-2 marker; lane 2 fibroblast alone, lane 3, tumor cell alone, lane 4, fibroblast and tumor cell co-culture. MMP-2 Fibroblasts exhibited a weak gelatinolytic band at 68 kDa, while tumor cells did not display any detectable gelatinolytic activities. In co-culture, tumor cells enhanced the gelatinolytic activity at 68 kDa. B. MT1-MMP expression in tumor cells. Tumor cells were immunostained with an MT1-MMP monoclonal antibody, and the resulting 60-kDa band is shown. (PDF 122 kb)

**Additional file 6: Table S1.** Immunostaining for emmprin and CD73 in the tumor cells and stromal fibroblasts performed on ten tumors of surgically resected or biopsied epithelioid sarcoma. CD73-close, indicates CD73 expression in stromal cells in proximity to the tumor cells; CD73-distant, indicates CD73 expression in stromal cells distant from the tumor cells. (PDF 19 kb)

## Data Availability

The original data sources and the dataset used in this analysis is available upon request to the corresponding author.

## Abbreviations

CD147; MMP: Matrix metalloproteinase
Emmprin: Extracellular matrix metalloproteinase inducer
MS: Mass spectrometry
PLA: In situ proximity ligation assay
siRNA: Small interference RNA

## References

[1] Biswas C, Zhang Y, DeCastro R, Guo H, Nakamura T, Kataoka H, Nabeshima K (1995). The human tumor cell-derived collagenase stimulatory factor (renamed EMMPRIN) is a member of the immunoglobulin superfamily. Cancer Res.

[2] Kataoka H, DeCastro R, Zucker S, Biswas C (1993). Tumor cell-derived collagenase-stimulatory factor increases expression of interstitial collagenase, stromelysin, and 72-kDa gelatinase. Cancer Res.

[3] Toole BP (2003). Emmprin (CD147), a cell surface regulator of matrix metalloproteinase production and function. Curr Top Dev Biol.

[4] Kanekura T, Chen X, Kanzaki T (2002). Basigin (CD147) is expressed on melanoma cells and induces tumor cell invasion by stimulating production of matrix metalloproteinases by fibroblasts. Int J Cancer.

[5] Tang Y, Nakada MT, Kesavan P, McCabe F, Millar H, Rafferty P, Bugelski P, Yan L (2005). Extracellular matrix metalloproteinase inducer stimulates tumor angiogenesis by elevating vascular endothelial cell growth factor and matrix metalloproteinases. Cancer Res.

[6] Nabeshima K, Iwasaki H, Koga K, Hojo H, Suzumiya J, Kikuchi M (2006). Emmprin (basigin/CD147): matrix metalloproteinase modulator and multifunctional cell recognition molecule that plays a critical role in cancer progression. Pathol Int.

[7] Grass GD, Toole BP (2015). How, with whom and when: an overview of CD147-mediated regulatory networks influencing matrix metalloproteinase activity. Biosci Rep.

[8] Muraoka K, Nabeshima K, Murayama T, Biswas C, Koono M (1993). Enhanced expression of a tumor-cell-derived collagenase-stimulatory factor in urothelial carcinoma: its usefulness as a tumor marker for bladder cancers. Int J Cancer.

[9] Sameshima T, Nabeshima K, Toole BP, Yokogami K, Okada Y, Goya T, Koono M, Wakisaka S (2000). Expression of emmprin (CD147), a cell surface inducer of matrix metalloproteinases, in normal human brain and gliomas. Int J Cancer.

[10] Muramatsu T (2016). Basigin (CD147), a multifunctional transmembrane glycoprotein with various binding partners. J Biochem.

[11] Guo H, Zucker S, Gordon MK, Toole BP, Biswas C (1997). Stimulation of matrix metalloproteinase production by recombinant extracellular matrix metalloproteinase inducer from transfected Chinese hamster ovary cells. J Biol Chem.

[12] Sun J, Hemler ME (2001). Regulation of MMP-1 and MMP-2 production through CD147/extracellular matrix metalloproteinase inducer interactions. Cancer Res.

[13] Kawakami T, Sameshima T, Hojo H, Koga K, Nakahara Y, Toole BP, Suzumiya J, Okada Y, Iwasaki A, Nabeshima K (2011). Synthetic emmprin peptides with chitobiose substitution stimulate MMP-2 production by fibroblasts. BMC Cancer.

[14] Stetler-Stevenson WG, Liotta LA, Kleiner DE (1993). Extracellular matrix 6: role of matrix metalloproteinases in tumor invasion and metastasis. FASEB J.

[15] Suzuki S, Sato M, Senoo H, Ishikawa K (2004). Direct cell-cell interaction enhances pro-MMP-2 production and activation in co-culture of laryngeal cancer cells and fibroblasts: involvement of EMMPRIN and MT1-MMP. Exp Cell Res.

[16] Koga K, Aoki M, Sameshima T, Hamasaki M, Egawa N, Seiki M, Toole BP, Suzumiya J, Nabeshima K (2011). Synthetic emmprin peptides inhibit tumor cell-fibroblast interaction-stimulated upregulation of MMP-2 and tumor cell invasion. Int J Oncol.

[17] Nishio J, Iwasaki H, Nabeshima K, Ishiguro M, Naumann S, Isayama T, Naito M, Kaneko Y, Kikuchi M, Bridge JA (2005). Establishment of a new human epithelioid sarcoma cell line, FU-EPS-1: molecular cytogenetic characterization by use of spectral karyotyping and comparative genomic hybridization. Int J Oncol.

[18] Aoki M, Koga K, Hamasaki M, Egawa N, Nabeshima K (2017). Emmprin, released as a microvesicle in epithelioid sarcoma, interacts with fibroblasts. Int J Oncol.

[19] Koga K, Nabeshima K, Aoki M, Kawakami T, Hamasaki M, Toole BP, Nakayama J, Iwasaki H (2007). Emmprin in epithelioid sarcoma: expression in tumor cell membrane and stimulation of MMP-2 production in tumor-associated fibroblasts. Int J Cancer.

[20] Sameshima T, Nabeshima K, Toole BP, Yokogami K, Okada Y, Goya T, Koono M, Wakisaka S (2000). Glioma cell extracellular matrix metalloproteinase inducer (EMMPRIN) (CD147) stimulates production of membrane-type matrix metalloproteinases and activated gelatinase a in co-cultures with brain-derived fibroblasts. Cancer Lett.

[21] Koyama-Nasu R, Takahashi R, Yanagida S, Nasu-Nishimura Y, Oyama M, Kozuka-Hata H, Haruta R, Manabe E, Hoshino-Okubo A, Omi H (2013). The cancer stem cell marker CD133 interacts with plakoglobin and controls desmoglein-2 protein levels. PLoS One.

[22] Tatsukawa R, Koga K, Aoki M, Koshikawa N, Imafuku S, Nakayama J, Nabeshima K (2016). Immunohistochemical demonstration of EphA2 processing by MT1-MMP in invasive cutaneous squamous cell carcinoma. Virchows Arch.

[23] Overall CM, Sodek J (1990). Concanavalin a produces a matrix-degradative phenotype in human fibroblasts. Induction and endogenous activation of collagenase, 72-kDa gelatinase, and Pump-1 is accompanied by the suppression of the tissue inhibitor of matrix metalloproteinases. J Biol Chem.

[24] Morandini AC, Savio LE, Coutinho-Silva R (2014). The role of P2X7 receptor in infectious inflammatory diseases and the influence of ectonucleotidases. Biom J.

[25] Gao ZW, Dong K, Zhang HZ (2014). The roles of CD73 in cancer. Biomed Res Int.

[26] Zhi X, Chen S, Zhou P, Shao Z, Wang L, Ou Z, Yin L (2007). RNA interference of ecto-5'-nucleotidase (CD73) inhibits human breast cancer cell growth and invasion. Clin Exp Metastasis.

[27] Hibino T, Sakaguchi M, Miyamoto S, Yamamoto M, Motoyama A, Hosoi J, Shimokata T, Ito T, Tsuboi R, Huh NH (2013). S100A9 is a novel ligand of EMMPRIN that promotes melanoma metastasis. Cancer Res.

[28] Gallagher SM, Castorino JJ, Wang D, Philp NJ (2007). Monocarboxylate transporter 4 regulates maturation and trafficking of CD147 to the plasma membrane in the metastatic breast cancer cell line MDA-MB-231. Cancer Res.

[29] Fukuoka M, Hamasaki M, Koga K, Hayashi H, Aoki M, Kawarabayashi T, Miyamoto S, Nabeshima K (2012). Expression patterns of emmprin and monocarboxylate transporter-1 in ovarian epithelial tumors. Virchows Arch.

[30] Xu D, Hemler ME (2005). Metabolic activation-related CD147-CD98 complex. Mol Cell Proteomics.

[31] Tang W, Chang SB, Hemler ME (2004). Links between CD147 function, glycosylation, and caveolin-1. Mol Biol Cell.

[32] Tang W, Hemler ME (2004). Caveolin-1 regulates matrix metalloproteinases-1 induction and CD147/EMMPRIN cell surface clustering. J Biol Chem.

[33] Egawa N, Koshikawa N, Tomari T, Nabeshima K, Isobe T, Seiki M (2006). Membrane type 1 matrix metalloproteinase (MT1-MMP/MMP-14) cleaves and releases a 22-kDa extracellular matrix metalloproteinase inducer (EMMPRIN) fragment from tumor cells. J Biol Chem.

[34] Zhao P, Zhang W, Wang SJ, Yu XL, Tang J, Huang W, Li Y, Cui HY, Guo YS, Tavernier J (2011). HAb18G/CD147 promotes cell motility by regulating annexin II-activated RhoA and Rac1 signaling pathways in hepatocellular carcinoma cells. Hepatology.

[35] Zhao P, Zhang W, Tang J, Ma XK, Dai JY, Li Y, Jiang JL, Zhang SH, Chen ZN (2010). Annexin II promotes invasion and migration of human hepatocellular carcinoma cells in vitro via its interaction with HAb18G/CD147. Cancer Sci.

[36] Berditchevski F, Chang S, Bodorova J, Hemler ME (1997). Generation of monoclonal antibodies to integrin-associated proteins. Evidence that alpha3beta1 complexes with EMMPRIN/basigin/OX47/M6. J Biol Chem.

[37] Wu XR, He XS, Chen YF, Yuan RX, Zeng Y, Lian L, Zou YF, Lan N, Wu XJ, Lan P (2012). High expression of CD73 as a poor prognostic biomarker in human colorectal cancer. J Surg Oncol.

[38] Liu N, Fang XD, Vadis Q (2012). CD73 as a novel prognostic biomarker for human colorectal cancer. J Surg Oncol.

[39] Yang Q, Du J, Zu L (2013). Overexpression of CD73 in prostate cancer is associated with lymph node metastasis. Pathol Oncol Res.

[40] Leclerc BG, Charlebois R, Chouinard G, Allard B, Pommey S, Saad F, Stagg J (2016). CD73 expression is an independent prognostic factor in prostate Cancer. Clin Cancer Res.

[41] Lu XX, Chen YT, Feng B, Mao XB, Yu B, Chu XY (2013). Expression and clinical significance of CD73 and hypoxia-inducible factor-1alpha in gastric carcinoma. World J Gastroenterol.

[42] Loi S, Pommey S, Haibe-Kains B, Beavis PA, Darcy PK, Smyth MJ, Stagg J (2013). CD73 promotes anthracycline resistance and poor prognosis in triple negative breast cancer. Proc Natl Acad Sci U S A.

[43] Turcotte M, Spring K, Pommey S, Chouinard G, Cousineau I, George J, Chen GM, Gendoo DM, Haibe-Kains B, Karn T (2015). CD73 is associated with poor prognosis in high-grade serous ovarian cancer. Cancer Res.

[44] Ren ZH, Lin CZ, Cao W, Yang R, Lu W, Liu ZQ, Chen YM, Yang X, Tian Z, Wang LZ (2016). CD73 is associated with poor prognosis in HNSCC. Oncotarget.

[45] Edlund K, Lindskog C, Saito A, Berglund A, Ponten F, Goransson-Kultima H, Isaksson A, Jirstrom K, Planck M, Johansson L (2012). CD99 is a novel prognostic stromal marker in non-small cell lung cancer. Int J Cancer.

[46] Byun HJ, Hong IK, Kim E, Jin YJ, Jeoung DI, Hahn JH, Kim YM, Park SH, Lee H (2006). A splice variant of CD99 increases motility and MMP-9 expression of human breast cancer cells through the AKT-, ERK-, and JNK-dependent AP-1 activation signaling pathways. J Biol Chem.

