# Supplementary figures and images for: CD73 complexes with emmprin to regulate MMP-2 production from co-cultured sarcoma cells and fibroblasts
Source: BMC Cancer. 2019 Sep 12;19:912. doi: 10.1186/s12885-019-6127-x (PMC6739984; doi:10.1186/s12885-019-6127-x)

**A**

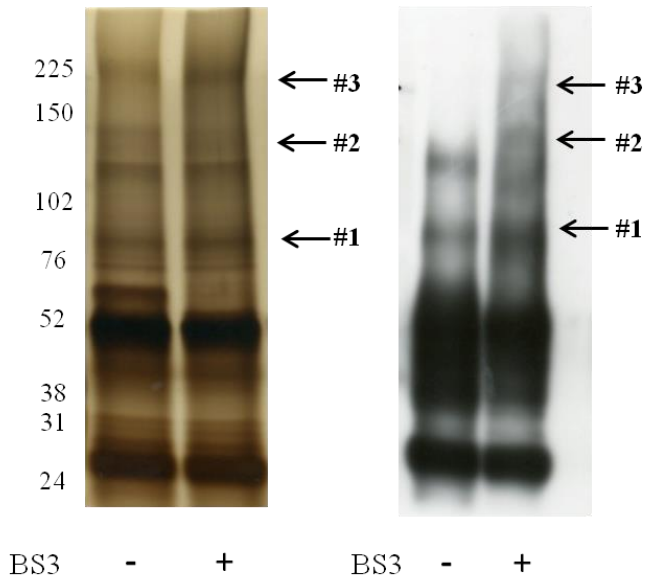

**B**

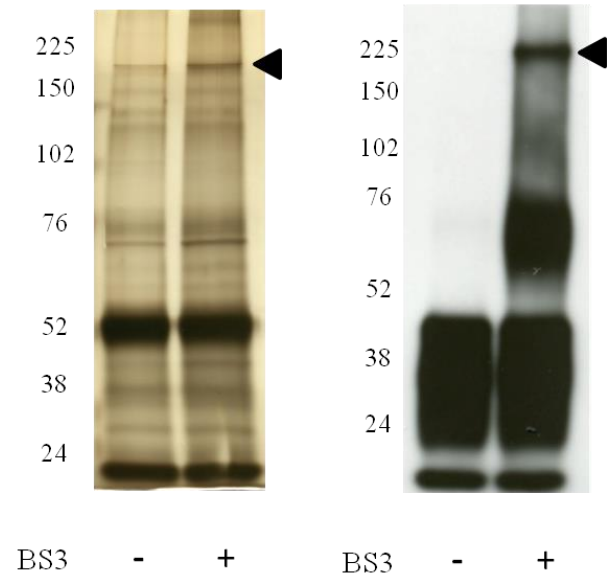

Supplement: Supplementary file 1 — Additional file 1:Figure. S1. Proteins isolated from membrane preps were subjected to silver staining in SDS-polyacrylamide gels (left) and immunoblotting (right). Mass spectrometry (MS) analysis was performed on the excised gel samples (identical molecular weight bands detected with anti-emmprin antibody in BS3 treated and BS3 non-treated samples). Three bands detected in the co-culture of tumor cells and fibroblasts shown in A (arrow #1, 75–100 kDa; arrow #2, 100–140 kDa; and arrow #3, 220 kDa) and a single band detected in tumor cells alone shown in B (arrow head, 220 kDa) were analyzed. (PDF 64 kb) [file 12885_2019_6127_MOESM1_ESM.pdf]

IB: Emmprin

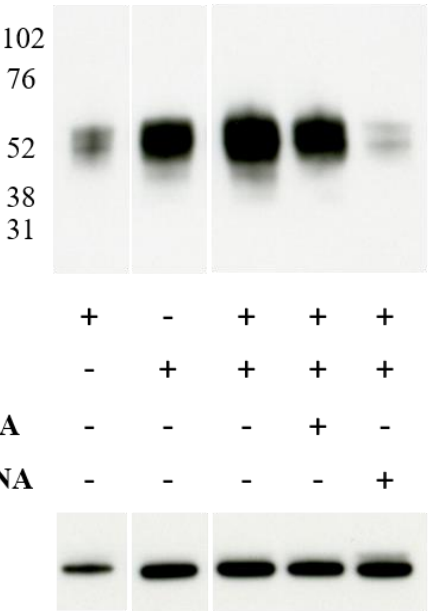

IB: MMP-2

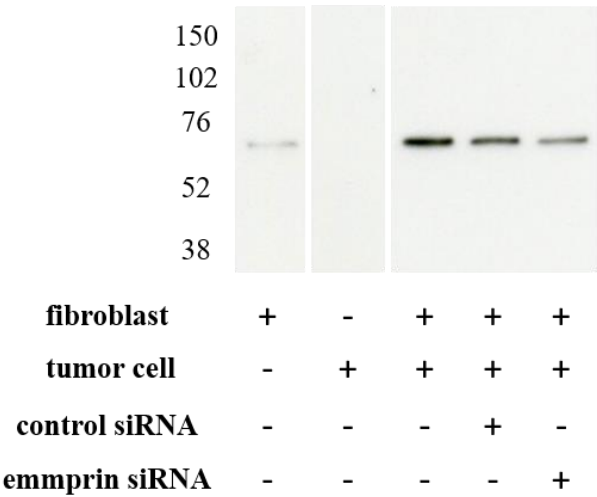

Supplement: Supplementary file 2 — Additional file 2:Figure. S2. Emmprin siRNA (0.02 pmol/μl) treatment causes knockdown of emmprin expression (40-60 kDa) in co-culture of tumor cells (FU-EPS-1) with fibroblasts (ST353i). Protein were extracted from a membrane preparation. Reduction of MMP-2 production in the conditioned medium was observed upon siRNA mediated emmprin knockdown of co-cultured cells. (PDF 45 kb) [file 12885_2019_6127_MOESM2_ESM.pdf]

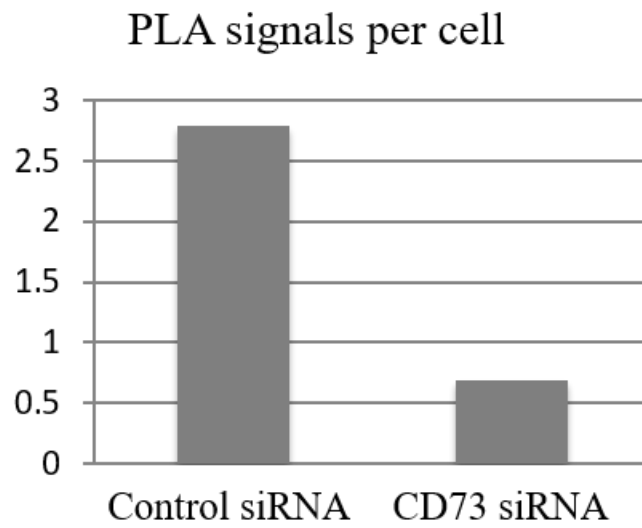

Supplement: Supplementary file 3 — Additional file 3: Figure. S3. Quantification of PLA signals was performed by Image tool analysis (Duolink). (PDF 14 kb) [file 12885_2019_6127_MOESM3_ESM.pdf]

**CD73-close**

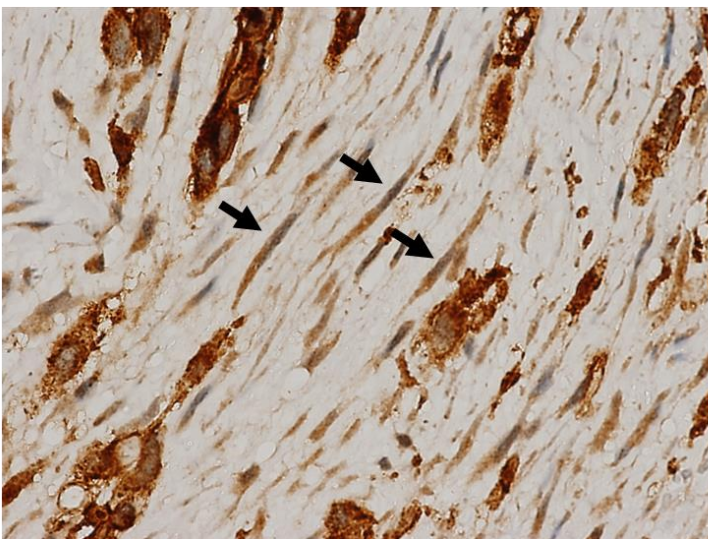

**CD73-distant**

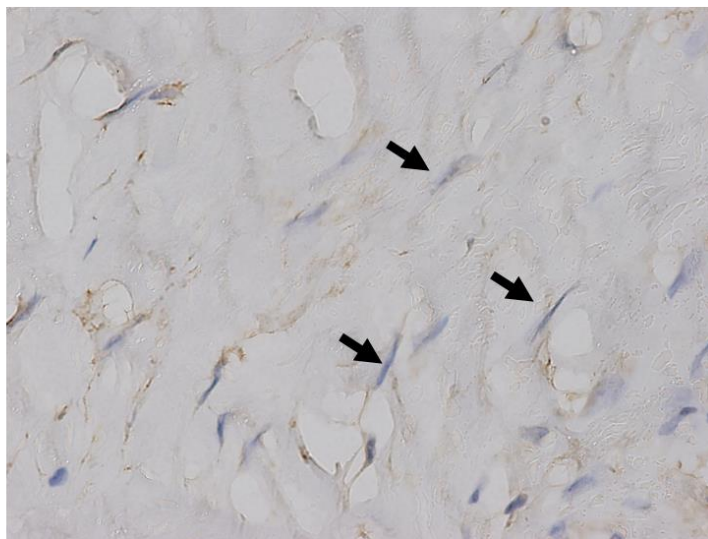

Supplement: Supplementary file 4 — Additional file 4: Figure. S4. Representative CD73 expression in the stromal fibroblasts (arrow). CD73-close, close to the tumor cells scale 3+; CD73-distant, distant from the tumor cells scale 1 + . (PDF 138 kb) [file 12885_2019_6127_MOESM4_ESM.pdf]

**A**

**Zymography**

Pro-MMP-2 ➡

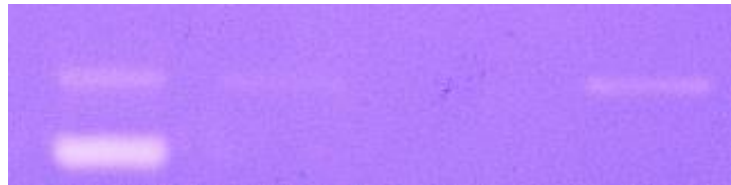

**Fibroblast**

+

-

+

**Tumor cell**

-

+

+

**B**

**IB: MT1-MMP**

(kDa)

150

102

76

52

38

24

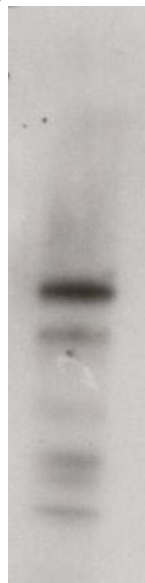

**Tumor cell**

Supplement: Supplementary file 5 — Additional file 5: Figure. S5. A. MMP-2 gelatinolytic activity in fibroblasts and co-culture. Gelatin zymography was performed with culture media collected on day 7 of culture. Bands at 68 kDa correspond to the pro-form of MMP-2. Lane 1, MMP-2 marker; lane 2 fibroblast alone, lane 3, tumor cell alone, lane 4, fibroblast and tumor cell co-culture. MMP-2 Fibroblasts exhibited a weak gelatinolytic band at 68 kDa, while tumor cells did not display any detectable gelatinolytic activities. In co-culture, tumor cells enhanced the gelatinolytic activity at 68 kDa. B. MT1-MMP expression in tumor cells. Tumor cells were immunostained with an MT1-MMP monoclonal antibody, and the resulting 60-kDa band is shown. (PDF 122 kb) [file 12885_2019_6127_MOESM5_ESM.pdf]
