# Supplementary material for: CD73 complexes with emmprin to regulate MMP-2 production from co-cultured sarcoma cells and fibroblasts
Source: BMC Cancer. 2019 Sep 12;19:912. doi: 10.1186/s12885-019-6127-x (PMC6739984; doi:10.1186/s12885-019-6127-x)
Supplement: Supplementary file 6 — Additional file 6: Table S1. Immunostaining for emmprin and CD73 in the tumor cells and stromal fibroblasts performed on ten tumors of surgically resected or biopsied epithelioid sarcoma. CD73-close, indicates CD73 expression in stromal cells in proximity to the tumor cells; CD73-distant, indicates CD73 expression in stromal cells distant from the tumor cells. (PDF 19 kb) [file 12885_2019_6127_MOESM6_ESM.pdf]

	age	location	tumor cells		stromal cells	
			Emmprin	CD73	CD73-close	CD73-distant
1	33	thigh	3+	3+	2+	2+
2	34	forearm	2+	3+	2+	2+
3	65	vulva	3+	3+	2+	1+
4	35	thigh	2+	3+	2+	2+
5	33	arm	3+	3+	3+	3+
6	68	forearm	3+	3+	3+	2+
7	34	thigh	2+	3+	3+	3+
8	61	shoulder	2+	3+	3+	3+
9	81	vulva	2+	3+	3+	3+
10	22	upperarm	3+	3+	2+	1+
